# “We’re led by stupid people”: Exploring Trump’s use of denigrating and deprecating speech to promote hatred and violence

**DOI:** 10.1007/s10611-023-10085-y

**Published:** 2023-02-09

**Authors:** Jace Valcore, Nicole L. Asquith, Jess Rodgers

**Affiliations:** 1 Denver Sheriff Department, Denver, CO USA; 2 Tasmanian Institute of Law Enforcement Studies, University of Tasmania, Hobart, TAS Australia

**Keywords:** Hate speech, Hate crime, Verbal-textual hostility, Political speech, Trump

## Abstract

In response to a call for criminologists to consider the impact of former President Donald Trump’s presumed criminality, we analyze verbal-textual hostility (VTH) in Trump’s campaign speeches. Politicians have particular power and reach with their speech and their use of VTH is an important part of the trifecta of violence. Using a framework informed by linguistic theory and previous analysis of hate speech in recorded hate crimes, we present the categories of deprecation and denigration, and discuss their relationship to domination. In context, these forms of VTH enhance and serve as precursors to more violent speech and acts.

In their 2021 essay for *The Criminologist*, Barak and Friedrichs called for criminologists to prove their relevance and explore the impact of former President Trump’s presumed criminality on American ideas of crime and criminal law. With this study we attempt to partially answer that call by focusing on Trump’s “promotion of white nationalism and hate crimes” through his political campaign speeches (Barak & Friedrichs, 2021, p. 8). We began this exploration by applying Asquith’s (2013) verbal-textual hostility (VTH) framework and identifying the most common categories of hostile speech that Trump employed. In a previous article, we analyzed and discussed three major categories: criminalization, domination, and expatriation (Valcore et al., 2021). In this article we analyze two unique categories of VTH that Trump commonly employed, denigration and deprecation, and discuss their relation to domination, which is explicitly white nationalist and white supremacist speech. The original intent of this study was to explore the connection between hateful rhetoric and hate violence, and we believe it can further contribute to understanding of the “trifecta of violence,” where the relationship between hateful rhetoric and state-sanctioned discrimination leads to increases in hate crimes and bias-motivated violence (Lenning et al., 2021), as well as the continuum of hate (Schweppe & Perry, 2021).

## Political speech

Persuasion is a defining characteristic of politics, and speech is the primary mode through which humans attempt to persuade one another to particular judgements or decisions (Martin, 2013). Rhetoric and oratory have long been described and recognized “as fundamental to the integrity of a healthy democracy” (Crines & Lehrman, 2016, p. 1). Free speech—and political speech, in particular—is afforded the highest, strictest levels of protection. Free speech in the United States (US) context, however, allows for the dissemination of even the most “ill-informed, repulsive and sometimes injurious views” because “the spiteful, the prejudiced and the plain small-minded”^1^ are provided an equal platform along with the truthful and wise (Martin, 2013, p. 3). As with contemporary marketplaces, the marketplace of ideas, in this respect, offers the most dangerous and polluting products for the lowest cost.

In a political campaign speech, the goal is to persuade the audience to certain attitudes and actions. The political persuader uses language to affect perceptions of knowledge, belief, value, and action. Unlike legal or legislative persuasion that typically require logic and rationality, political campaigns operate in a world where it is not required for “every statement be logically defensible” (McBath & Fisher, 1969, p. 17). Scholars of political rhetoric assume that political speakers need a unifying argument that resonates with both the immediate and wider audiences in order to be successful (Crines & Lehrman, 2016). They further assume that political speakers use language strategically to win support, establish a positive image, and assert specific beliefs and policy solutions. The language of politicians is used to name and label social problems and to identify and define friends and foes (Schulz, 2015). Political arguments make judgments about the dimensions and limits of human association, including the identification and drawing of lines between in and out groups. As Martin (2013, p. 6) explains, “In that respect, there is always a trace of violence (whether real or implied) that surrounds political rhetoric because ‘matters of principle’ invoke the limits of what is thinkable and do-able.” Ideally though, political campaigns provide a reinvigorating conversation among candidates, the media, and the public that support democratic institutions and practices (Hart et al., 2013). Research has shown that citizens dislike negativity in campaigns and often seek candidates who occupy centrist or “middle-ground” positions (Parry-Giles & Samek, 2014), though more recent successful campaigns, such as that of Donald J. Trump, certainly suggest that this is not a universal rule.

## Politicians and hate speech

Politicians have social influence, and their use of hostile and hateful speech can have considerable effects (Benesch et al., 2020). Perry et al. (2020) uses the example of the Rwandan genocide to demonstrate the impact of incitement from politicians. They note that decades of speech against Tutsis via public radio and politicians’ use of anti-Tutsi rhetoric contributed to the incitement of violence, which included politicians publicly provoking supporters into mass murder (Perry et al., 2020). The role of politicians and hate speech in genocide and civil war is also noted in the Former Yugoslavia and Sudan (Piazza, 2020a) and, of course, the genocide of the Jewish people in Europe in the 1930s and 1940s. One example is that of *Der Stűrmer* under the editorialship of Julius Streicher. Streicher was a member of the Nazi party and parliament during Nazi rule and the newspaper’s roles in inciting hatred against Jews was perceived as distasteful by many middle-class Germans, but it facilitated a common language of hatred against Jews, including the national practice of denigrating outsiders and deprecating Germany for allowing Jews to thrive and succeed whilst they languished (Bytwerk, 2001; Showalter, 1983).^2^ In Turkey, Perry et al. (2020) found that hate rhetoric from politicians aimed at a specific group may correspond with a rise in hate crimes against that group. Examining counts of domestic terrorism and use of hate speech in the rhetoric of major political parties for 135–163 countries from 2000 to 2017, Piazza (2020a) argued that hate speech by politicians increases political polarization and that this, in turn, increases domestic terrorism. The rate of domestic terrorism increases almost nine times when politicians often use hate speech in public statements. In Nigeria, hate speech during election campaigns was suggested to be a major driver of violence around elections (Ezeibe, 2020).

Trump’s explicit use of hateful speech—what was previously often coded language (McIlwain & Caliendo, 2011) — was not original or unprecedented. Piazza (2020b) noted, “President Donald Trump is not the only world leader who is accused of publicly denigrating people based on their racial, ethnic or religious backgrounds” (para. 2). But as has been widely documented, Trump violated numerous democratic and political norms in both the delivery and content of his speeches (see, e.g., Ross & Rivers, 2020; Jamieson & Taussig, 2017) and was the only US President confirmed through empirical analysis of his campaign speeches to be a right-wing populist (Çinar et al., 2020).

Populism is defined by emotional appeals to the people, anti-elitism, and the exclusion of out-groups who are routinely blamed and scapegoated for perceived grievances and social ills (Aalberg & de Vreese, 2016). Right-wing populists target political elites and make ethnic and racial-based claims, in contrast to left-wing populists, like former Presidential candidate Bernie Sanders, who target economic elites and focus on class-based concerns (Çinar et al., 2020). In fact, quantitative analysis by Çinar et al. (2020) showed that no other comparable candidate of either major US party has ever approached the level of negativity and vitriol toward racial/ethnic minorities that Trump did. Trump’s speech during his campaigns and presidency was defined by informality, anti-intellectualism, insults, self-aggrandizement, demonization of all who oppose him or disagree with him, and a “Manichean, apocalyptic rhetoric of demise and deliverance” (Jamieson & Taussig, 2017, p. 641; Ross & Rivers, 2020). While his Manichean mind-set is not unique, his right-wing populism that involved attacks on democratic institutions and blatant appeals to racial prejudice was norm-shattering (Çinar et al., 2020; Jamieson & Taussig, 2017).

Presidents are uniquely positioned to influence national discourse (Stuckey, 2020), and as Aalberg and de Vreese (2016) argued, the effects and consequences of populist messages need to be explored. While not criminal in nature according to US constitutional law, Trump’s verbal hostility has been connected to acts of violence and increases in the expression of prejudice among his audience (Gonzalez-Gorman, 2018; Newman et al., 2018), including the incitement to violence toward protesters who dared to attend his rallies (Schreckinger, 2016; White, 2016). Trump seemed to revel in the “power of the Presidency to hurt”; his vitriol and direct calls for violence circulated among those with pre-existing prejudices and encouraged them to take violent action (Stuckey, 2020). Through time series analysis Fortunato et al. (2022) showed that Trump’s nationalistic agenda emboldened right-wing extremist groups and individuals, resulting in abrupt increases in violent attacks and domestic terrorism. Thus, it is imperative for criminologists and legal scholars to examine and understand the rhetoric and forms of speech used by populist leaders like Trump.

We previously identified and labeled populist and racist rhetoric that Trump often utilized to appeal to his target audience of white Americans as a new VTH category of Domination (Valcore et al., 2021). Trump’s dominating speech was intended to stoke fear about immigrants and racially minoritized groups, and incite his audience to action, even violence (Valcore et al., 2021). His two most popular campaign slogans, “Make America Great Again” and “America First” were emblematic of his white nationalist and populist beliefs (“America First” was also a slogan of the Ku Klux Klan in the 20^th^ century). As any good campaign slogan must do, these phrases provided a clear statement of the goals and purpose of his campaign that allowed his audience to identify and align themselves with him (Borgstrom, 1982).

## Theoretical framework

To understand the power and effect of Trumpian political speech, it is necessary to draw from the theoretical framings of both political science and linguistics. Of the latter, Austin (1980 [1955]) proposed that there are three forms of speech: locutionary, illocutionary, and perlocutionary. In the context of the study of VTH, illocutionary speech acts are the most important, as they have force and a specific goal, such as ordering, warning, and informing. Perlocutionary speech acts hope to achieve something in the saying, such as convincing, persuading, inciting, or misleading. Analyzing verbal and textual hostility in hate crime, Asquith (2007, 2008, 2009, 2010, 2013) argues the majority of what is categorized as “hate speech” is illocutionary. This means that it achieves its aim—of warning, threatening, silencing—in being said, not necessarily or only as a consequence of being said.

In order to bypass the seemingly straight-forward proclamation that “Congress shall make no law … abridging the freedom of speech …”, Asquith and others have conceptualized illocutions as action, not pure speech (see, for example, Butler, 1997; Langton, 1993; Matsuda et al., 1993). This is specifically relevant for verdictives (exercise of judgement) and exercitives (exercise of power) (Austin, 1980 [1955]), which Langton (1993) recategorized as *authoritative illocutions*. In VTH, verdictives name individuals within a hierarchy according to their distance or propinquity to dominant representations of the body politic. Exercitive illocutions warn, order, and command, and most commonly require an authorized force in order to be effective. Unlike verdictive illocutions that are “… temporally present or an assessment of the past, exercitives are statements about how the future should look: an advocacy or threat of things to come” (Asquith, 2009, p. 164). Thus, exercitives coming from an authorized individual, such as elected politicians or political candidates, are more capable of acting on audiences than verdictives, which gain their power through the incitement of others to act.

Verbal-textual hostility is a socially authorized social artefact. Social power is invested in all speech acts before their speaking (Bourdieu, 1991), which necessitates that their efficacy is not dependent on the power or usage of specific words, but the social context in which they are spoken. Whose speech counts, who is allowed to speak, and who is silenced is based on social and political authorization. Authoritative speech requires social recognition of a speaker and a topic that is perceived as legitimate (Langton, 1993). Commonly, this social authorization is denied for those who are targets of VTH and retained by those who use “words to wound” (Matsuda et al., 1993). Through the analysis of the harms generated from illocutionary speech acts it is acknowledged that words wound in their saying, *and* as a consequence of the saying. Importantly, as Asquith suggests, “… understanding the consequential—or perlocutionary—effects enables us to understand the process of incitement and the power of infecting others’ minds—and perhaps their actions” (2010, p. 102). The theoretical framework needed to acknowledge the individual harms of VTH, while also recognizing the institutionally-bound and socially contingent aspects is provided by the critical race theory informing Matsuda et al.’s (1993) collection, *Words that Wound*, as well as the linguistic theory developed by Austin (1980 [1955]), Bourdieu (1991), Butler (1997), and Langton (1993).

## Methodology

We used Asquith’s (2013) Verbal-Textual Hostility (VTH) framework to first identify, and then Critical Discourse Analysis (CDA) to analyze, verbal-textual hostility in political speeches delivered by Donald J. Trump as a candidate and President of the United States (van Dijk, 1997; Wodak, 2001). The VTH framework allowed us to see how Trump’s speech aligned with speech acts reported during hate crimes and hate incidents, and to determine any new categories within Trump’s norm-shattering political rhetoric that can further our understanding of the connection between speech and violence. In this light, the VTH framework is useful for exploring the continuum of hatred (Schweppe & Perry, 2021). Van Dikj’s (1997) CDA framework for analyzing racism and prejudice provided the scaffolding for our analysis of Trump’s words in context. CDA enables us to consider socio-historical roots, institutional factors, social and political context, as well as force and effects of verdictive and exercitive speech acts.

### Sampling

We collected the transcripts of all of Trump’s campaign and rally speeches from the time of his candidacy announcement for President until the 2018 midterm elections (June 16, 2015-November 6, 2018). These were downloaded from The American Presidency Project, a non-profit and non-partisan database of presidential documents hosted by the University of California, Santa Barbara. From the specified period, there were a total of 90 speeches, with a corpus of 364,810 words.

### Coding

We refined our coding framework through multiple stages of coding, team discussion, and inter-rater reliability testing. First, we each coded the same three speeches using the revised Verbal & Textual Hostility typology developed by Asquith (2013). Our first round of coding found that there were hostile speech acts that did not align with the pre-existing framework and needed additional categorizing:

1. Denigration: stupid, loser, ugly, and silly (previously coded by Asquith (2013) as “other”)
2. Deprecation: criticism or ridicule in order to make claims about political action (e.g., “our country is a mess”), and
3. Domination: racist, nativist, and white supremacist speech.

We conducted another round of coding where each author coded the same three additional speeches (for a total of six co-coded speeches), which represented 10% of the total sample word count. Using the refined and expanded typology, the inter-rater agreement for each theme was 97%. One author then coded the remainder of the sample. We then each analyzed one of the three new themes, including the identification of child themes.

### Analysis

We completed coding and analysis using NVivo. In addition to qualitative coding, we used automatic quantitative analysis tools in NVivo to identify the most common or frequently used forms of speech. We ran Queries and Node Matrices for specific dates and locations—such as the date of Presidential candidate nomination and election, and between Republican and Democrat states (based on the 2012 election)—to identify any significant patterns or changes in VTH around these parameters. We found no significant patterns apart from the ebbs and flows of topics, phrases, and issues as the political campaign proceeded and after Trump was inaugurated. All quotes are attributable to Donald J. Trump unless stated otherwise.

## Findings

In applying the VTH framework to Trump’s campaign speeches, three new categories of speech were identified that expressed anger, resentment, and other forms of hostility: deprecation, denigration, and domination. One of these themes—denigration—was previously identified by Asquith in her revised taxonomy (2013), but only as a cluster of speech acts that exceeded the other defined categories of hate speech and that was labeled “Other.” Perhaps as an artefact of the data, or of the categories of hate crime recognized in crime reporting, these “other” speech acts coalesced in Trumpian language as ableist and ageist denigration.^3^ In addition to the newly recategorized speech act of denigration, we found two authoritative illocutions rarely used in hate speech incidents, perhaps due to the exercitive force needed to empower both domination and deprecation. Domination, as noted above, was analyzed separately in Valcore et al. (2021), but its connection to deprecation and denigration is explored here.

### Deprecation

Trump was a unique politician in many ways, some of which endeared him to US voters who felt disenfranchised. Almost without equivalence before Trump—but now a familiar tool in political speech—deprecation was commonplace throughout his campaign speeches. To deprecate means to “plead earnestly against; to express an earnest wish against (a proceeding); to express earnest disapproval of (a course, plan, purpose)” (Oxford English Dictionary, 2021), “to belittle, disparage, or to withdraw official support for” (Merriam-Webster Dictionary, 2021). While seeming to have no immediate and obvious relationship to VTH, we show how deprecation is used as a political tool, and explore the relationship to harm in the discussion. In this context, deprecation refers to statements where Trump claims that the United States is performing poorly, is less than other countries, is not the best, or is inadequate. For example,

They're tired of a country that has horrible trade deals, that has no borders, that has taxes that are through the roof, highest taxed nation just about in the world, that has regulations that don't allow you to start a business and destroy your business if you do start. (New York City, New York – July 16, 2016)

We have enough problems. We've got big problems. We have problems like you wouldn't believe. (Colorado Springs,  Colorado – October 18, 2016)

As with so much to do with Trump, hyperbole and exaggeration—rather than earnestness—enhances the power of his deprecation. In the context of deprecation, common topics included the economy, labor force and production, infrastructure, trade deals, military strength, and border security. Biegon (2019) argued that Trump’s electoral success was aided by the rhetorical exploitation of declinist themes, as seen in these deprecating comments:

Today, Detroit has a per capita income of under $15,000 dollars, about half of the national average. 40 percent of the city's residents live in poverty, over two-and-half times the national average. The unemployment rate is more than twice the national average. Half of all Detroit residents do not work. (Remarks to the Detroit Economic Club – August 8, 2016)

Home ownership is at its lowest rate in 51 years. (Remarks to the Detroit Economic Club – August 8, 2016).

And in many cases, they're working two and three jobs, OK? But they're making less money now than they made 18 years ago. And those stats come right out, real wages. They're working harder, they're getting older. (Wilmington, North Carolina – August 9, 2016)

Our national debt has doubled in eight years, and our infrastructure throughout our country is crumbling, bad shape. Our airports, our roads, our bridges, our tunnels, our schools, our hospitals. (Springfield, Ohio – October 27, 2016)

Biegon (2019, p. 525) states there is a discursive element to “decline” as

… a complex, contested political phenomenon, which can be understood in a number of ways—from the “high politics” of global leadership to more mundane concerns experienced by the US population; from deteriorating hegemonic legitimacy and the perceived loss of status to dwindling economic output, deindustrialization and associated social ills.

Concerns with loss of status and hegemonic legitimacy echo strongly in Trump’s repeated discussion of winners and losers, which he sees a strict binary necessary for defining success (Biegon, 2019; see also, Çinar et al., 2020; Jamieson & Taussig, 2017)

We don't win at any level with anything. (Green Bay, Wisconsin – August 5, 2016)

Our country doesn't win anymore. We don't win anymore. (Colorado Springs, CO, October 18, 2016)

According to communication scholars, deprecation is commonly used to frame the speaker as a savior (Biegon, 2019; Gamsa, 2017), which is how it was applied by Trump. While he framed America as currently losing, he pitched himself as the one to turn it around:

We need — we need somebody — we need somebody that literally will take this country and make it great again. We can do that.

We will make America wealthy again.

We will make America strong again.

We will make America safe again.

And we are going to make America great again. (New York City, New York – June 16, 2015)

By using ‘we’ in the summation and plans to restore the country, Trump simultaneously interpellated the audience and implicitly presented himself as the solution to the audience’s perceived woes. Deprecation’s strength is in its reference to the failure of past, current, and possible future leaders; albeit, at times, an implicit authoritative illocution.

Deprecation is an outlier in VTH in part because what we know of this speech act is largely linked to the psychological concept of self-deprecation. Self-deprecation “is widely understood within social psychology and popular culture as a form of self‐talk that reflects a cognitive state, such as low self‐esteem or negative self‐regard” (Speer, 2019, p. 806). Self-deprecation involves the disparagement of one’s own efficacy (Owens, 1994). Feelings of self-blame may be associated with self-deprecation (Owens, 1993), which is linked to depression and anxiety (Bakhtiari et al., 2017; Owens, 1994; Speer, 2019), and is a form of self-sabotage leading others to believe the negative statements (Speer, 2019).

Trump’s deprecation of the nation may have negative effects on the esteem of the audience, but it is not without utility as a communication practice. Speer (2019) identified a disconnect between the linguistic and psychological analysis of self-deprecation, noting their contrasting perspectives have not been reconciled. She argued that self-criticism and self-enhancement can simultaneously exist and “individuals can enhance their present selves by disparaging their past selves” (Speer, 2019, p. 807). This is clear in Trump’s use of deprecation—he exploited the human tendency to disparage and persuaded his audience that he could do better and fix the numerous failings of past administrations. Disparaging the past self, that is, the past nation/government, allowed Trump to place the blame on predecessors who, for instance, partnered in international trade deals:

Erie, has lost one in three manufacturing jobs. You know that, all I—you do—I flew over, you're looking at the plants, plants that 25 years ago, 20 years ago, 15 years ago, some two years ago, were vibrant. (Erie, Pennsylvania – August 12, 2016)

This state has lost more than 40 percent of its manufacturing jobs since Bill Clinton signed NAFTA, and it's lost one in four manufacturing jobs since he put China into the World Trade Organization, both deals supported by Hillary Clinton. Horrible deals, destructive deals, what those deals have done to our jobs and to our country. (Jackson, Mississippi – August 24, 2016)

Flaws in trade deals were made more tangible for the audience by linking them to employment:

Ohio has lost nearly 1 in 3 manufacturing jobs since NAFTA, and nearly 1 in 4 manufacturing jobs since China entered the World Trade Organization. (Wilmington, Ohio - September 1, 2016, Canton, Ohio – September 14, 2016)

According to the Economic Policy Institute, Michigan ranks first for jobs lost as a share of workforce due to the trade deficit with TPP members, and the country lost 740,000 manufacturing jobs as a result of that deficit last year. (Novi, Michigan – September 30, 2016)

This foments negative perspectives of previous governments and his opposition candidate. Here, the deprecation also induces a clear “other” for comparison, namely China in this context. As noted by Valcore et al. (2021), nationalist discourses focus on the threat to employment and resources from the other, and fear and hatred of the other is a key element of right-wing populism.

Gamsa (2017) found self-deprecation used in reference to the nation and its people by Lenin in Russia and the Chinese Communist Party (CCP) and Nationalist Party (NP) in China. Critiquing Russian populism in 1898, Lenin stated that ideas of this previously progressive movement now only embodied “stagnation and Asiatic backwardness” (Lenin, 1960–1970 in Gamsa, 2017, p. 404). In 1912, he saw China’s economic program as another form of populism criticizing that and stating that Russia was “undoubtedly an Asian country and, what is more, one of the most benighted, medieval and shamefully backward of Asian countries” (Lenin, 1960–1970 in Gamsa, 2017, p. 404). Reference to the people of China as Zhina / Zhinaren (Chinamen) by the Japanese (and utilized in this manner by various Chinese literary figures) was seen to signify China as a backwards country or the Chinese people as victims. Gamsa (2017, p. 413) states the CCP drew “massively on this rhetorical resource” to emphasize the humiliation China had suffered, but also by the NP to position themselves as saviors from the humiliation enacted by foreigners. Government efforts towards state remembrance of humiliation suffered in the hands of foreigners was very important for nationalism and faith in the government (Cohen, 2003). Both these examples draw on a defined “other” as a threat to the self, while deprecating the self, to assert the strength of current or campaigning governments.

Another topic common in the deprecation theme was military efficacy and strength, seemingly employed as a tool to warn his audience of current or impending national humiliation. Trump claims both that the US military is weak and outdated, and that ongoing military campaigns across the globe are negatively impacting homeland security.

Right now, we have the smallest air force since 1947, the smallest army since 1939, and one of the smallest Navies since 1917. (Greenville, North Carolina – September 6, 2016)

Our Army is the smallest it has been since before World War II. (Phoenix, Arizona – October 29, 2016)

We're spending $6 trillion on wars in the Middle East while our own country falls into total disrepair. (Springfield, Ohio – October 27, 2016)

They've dragged us into foreign wars that have made us less safe. (Orlando, Florida – November 2, 2016)

Our rivals no longer respect us. In fact, they are just as confused as our allies, but an even bigger problem is that they don't take us seriously anymore … Our enemies are getting stronger and stronger by the way, and we as a country are getting weaker. Even our nuclear arsenal doesn't work. (Washington, DC – March 21, 2016)

Trump makes false claims and notes perceived failures of past leadership while invoking the “other” as stronger and more powerful in order to instill fear and garner support. Describing perceived failures of previous leadership and making claims for improvement is hardly unique to Trump, but others noted that Trump’s speech was “unusually bleak” (Biegon, 2019, p. 526) and that his rhetoric “disrupted political and discourse norms” (Jamieson & Taussig, 2017, p. 649). Trump used these themes to position others as a threat to American greatness, and himself as the savior (Edwards, 2018; Jamieson & Taussig, 2017; Kiely, 2017), which forms part of his signature rhetorical style (Jamieson & Taussig, 2017) and aligns him with right-wing populist and nationalist leaders.

Deprecation of the nation was a useful political tool for Trump as he imbued it with elements of domination in order to unite the audience in support for his candidacy. Notably, deprecation was not a common theme in Trump’s speeches post-election; as President, it may be assumed that Trump became responsible for the state of the nation and could no longer utilize deprecating speech to assert the need for his leadership.

### Denigration

In the community (1995–2000) and police reports (2003–2007) that underpin her original and revised taxonomy of verbal-textual hostility, Asquith (2008, 2013) analyzed hate speech in hate crime. The datasets she accessed only related to homophobic, racist, Islamophobic and antisemitic hate crimes. In those datasets, many speech acts were recorded as “other” speech acts but the underlying pattern of those “other” speech acts was opaque until her taxonomy was applied to Trumpian political speech. Denigration, according to the *Oxford English Dictionary* (*OED*, 2022), means to “blacken” the character of a person, and disturbingly, when written with a hyphen—de-nigration—means to “whitewash.” With its clear linguistic links to a racial order, denigration may be dismissed as yet another form of racism given its Latin and French etymology to dusty, sooty, black and blacken. However, its earlier form from the sixteenth century, also links this speech act to mental illness and to the darkening and obscuring of “the spirit and sences” (Stubbes, 1583, cited in *OED*, 2022).

Whether ableism is used to deepen the offence of racism, or vice versa, there is a eugenicist link between the hated bodies of others (Thorneycroft & Asquith, 2021b). While most denigration goes unnoticed because it is embedded in social and political rhetoric, its foundation lies in the revulsion of bodies and minds that fail to meet the standards of those capable of speaking authoritatively on what and whose bodies and minds matter. Çinar et al. (2020) notes, in their comparative analysis of candidate speeches over time, that Trump’s denigration of other candidates was abnormally extreme, which enabled Trump to forge alliances with voters by pitching everyone else as “blackened”, “stained”, “sullied” (*OED*, 2022) and, as such, their common enemy.

In Trumpian speech acts, he links ableist terms (such as failure, loser, and fools, stupid and moron, and weak and tired) to the inefficacy of his political opponents, other countries, and racial and religious minorities. He also framed his adversaries as nasty, vicious, and horrible. In a single speech delivered in October 2016 in Colorado Springs, six percent of the corpus was coded as denigration. Importantly, most of his denigrating speech acts came in the early days of his campaign and by November 2016, he rarely deployed this form of VTH to make his point. It is unclear from the corpus whether denigrating rhetoric was removed from his arsenal as an explicit campaign choice, or whether he found other forms of VTH to be more powerful.

While Trump deployed denigration in his attacks against immigrants and immigration—such as when he stated that “… they're [Mexico] not sending their best … They're sending people that have lots of problems” (NYC, New York, June 16, 2015)—for the most part he preferred to criminalize immigrants as rapists and thugs (see Valcore et al., 2021). The main target for his denigration was Hilary Clinton and other political opponents:

he's [Biden] not a very bright guy. (Doral, Florida – July 27, 2016)

she [Clinton] will be a disaster for our country, a disaster in so many other ways … And you see what bad judgment she has. She has seriously bad judgment. (Phoenix, Arizona – August 31, 2016)

The Hillary Clinton campaign is so small, so petty, so tired. (Prescott Valley, Arizona – October 4, 2016)

Anthony Weiner is a proven loser. (News Conference in Doral, Florida – July 27, 2016)

Trump also reserved his denigrating comments about intelligence (or lack thereof) to his predecessors, and more generally to the American government and its decision makers.

I mean, you looked at Bush, it took him five days to answer the question on Iraq. He couldn't answer the question. He didn't know. I said, "Is he intelligent?" (New York City – June 16, 2015)

We're led by stupid people. (Doral, Florida – July 27, 2016)

They are dancing in the streets of Iran saying how stupid the Americans are. (Green Bay, Wisconsin – August 5, 2016)

Congratulations, folks. I hope you're doing well. OK? I hope you're doing well … But you know what? You can't do well. When you have a government that is so stupid, that is so—that is so incompetent… (Erie, Pennsylvania – August 12, 2016)

The man who killed her [Sarah Root] arrived at the border, entered Federal custody and then was released into the US, think of it, into the US community under the policies of the White House Barack Obama and Hillary Clinton. Weak, weak policies. Weak and foolish policies. (Phoenix, Arizona – August 31, 2016)

There's no common sense, there's no brain power in our administration by our leader, or our leaders. None, none, none. (Phoenix, Arizona – August 31, 2016)

The architect of Obamacare, Jonathan Gruber, admitted that the whole sales pitch was a lie – he called it the "stupidity of the American voter." But the only stupidity was the stupidity of our leaders who passed this disaster into law … (in Prescott Valley, Arizona - October 4, 2016)

The only stupidity was that incredible stupidity shown by our politicians when they forced this bill through over the furious objection to many politicians. (Springfield, Ohio – October 27, 2016)

It is easy to dismiss denigration as the rhetoric of a school-yard bully. However, the power of denigration comes in making its target seem smaller, less intelligent, less attractive, and as such, less capable of leading the nation. In framing his opponents as smaller, weaker, and inept, and deprecating his opponents’ actions and the nation itself, Trumpian political speech, again, sets up the “bait and switch” to laud his capacity and intelligence as the answer to the country’s supposedly damaged reputation.

### Denigration and domination

Trump’s deprecating speech was implicitly nationalist, while his denigrating speech was often explicitly so. The following quotes were coded for both denigration and domination, which makes the connection between denigrating, ableist speech and right-wing nationalism (domination) clear.

You know, the politicians say, you'll never, ever be able to get Mexico to allow you to build a wall. I say trust me. Now, they don't say that anymore. Now they say, they won't put the money up to build a wall. So easy. Mexico—our trade deficit is massive, massive. They make a fortune off the stupidity of the United States. Mexico will 100 percent—you hear—100 percent pay for the wall, 100 percent. (Erie, Pennsylvania – August 12, 2016)

We're not going to be the stupid people anymore. We're not going to be the stupid, weak people anymore. We are going to rebuild our country. Our country is going to be a country where you can be proud of again. We're going to use American steel, we're going to use American labor, we are going to come first in all deals. … We're not going to make the trade deal where we come in fifth and sixth and seventh and other countries laugh at our stupidity. We are going to build great companies. We are going to expand companies that are now doing poorly. (Ocala, Florida – October 12, 2016)

Attack after attack, including the recent terror strikes in New Jersey, New York, Minnesota, as well as the mall shooting in Washington, were made possible by our extremely open immigration system that's meant only to protect fools, and we're not fools. (Ocala, Florida – October 12, 2016)

Here, Trump makes repeated claims about “stupidity” and loss of international status in order to gain support for his nationalist and racist “America First” agenda, including the building of a wall on the Southern border to prevent migration by non-white “others.” Trump is claiming that the country has been damaged by globalization and diversification, and that in order to be “proud,” borders must be closed and international partners must be discarded.

## Discussion

Both denigrating and deprecating speech allowed Trump to paint himself as the only answer and the savior of the country. Çinar et al. (2020) argued that Trump’s rhetoric is increasingly common among populist leaders globally, who like Trump, blame globalism and democratic governments for turning their countries into “losers” (Biegon, 2019). The anti-trade sentiment, economic woes, and perceived military weakness lamented by Trump are a common thread in right-wing nationalist discourses and have been utilized in Brexit campaigning (Schmidt, 2017: Wilson, 2017), in the US 2006 midterm elections, where Mexican migrants were described as a threat to jobs and resources (McIlwain & Caliendo, 2011), and historically, in the Nazi Party’s policy for German protectionism (Anheier, 1997).

Both denigration and deprecation were almost exclusively employed during Trump’s campaign, and rarely appeared after his election, suggesting that the two forms of hostile rhetoric are useful for gaining support, but not maintaining it once in office. As noted by Lenning et al. (2021), these speech acts are part of a trifecta of violence, which deploys “violent ideology, violent policy, and violent actions” to undermine the political capital of those he opposed, along with those he demonized, criminalized, and terrorized. In this respect, while denigration and deprecation in isolation may be perceived as *just* rhetoric—albeit, relatively new political speech—when these speech acts are considered within the wider contexts of Trump’s verbal and textual hostility, the links between deprecation, denigration, and domination become transparent. In a similar argument Asquith (2013) has made in relation to the use of profanity in verbal-textual hostility, deprecating and denigrating speech acts are enhancers to the violent ideology espoused by Trump during campaigning and once in office. Alone these speech acts may appear inconsequential and relatively harmless. In context, we can identify the role they play in not only enhancing, but also as precursors to more violent speech. Importantly, for disabled people, the normalization of ableist hate speech in political discourse is as equally problematic now as it was in the early years of the Third Reich, and points to a reductionism, whereby antagonists to Trump’s plans are branded as “crips” (Thorneycroft & Asquith, 2021a).

### Deprecation

It is rare for a motivated hate crime offender to put themselves down as a means of causing harm to their target. However, as noted in many cases of extremist violence, the use of deprecation to enliven action—perlocutionary effects—against the other can be found in their manifestos and social media posts (see, for example, the manifestos of the Christchurch Mosque killer and, more recently, of the Buffalo Tops Supermarket killer). While not identified in earlier research on hate speech in hate crime (Asquith, 2008, 2013), deprecation may be a precursor to more targeted violent speech, and act as a provocation and incitement to addressees and bystanders rather than words that wound the targets of this speech/text. Deprecation is a perlocutionary message and permission to hate not because of some characteristic of the hated other, but for what has presumably been done by the hated other to the safe, clean, Arcadian, white world the speaker cherishes.

Though benign in comparison to the explicitly racist and violent speech employed in Trump’s dominating, criminalizing, and expatriating speech (Valcore et al., 2021), deprecation expresses the same fear of lost status and economic distress found among reactive/defensive hate crime offenders (McDevitt et al., 2002). Defensive hate crimes accounted for 25% of all reported hate crimes analyzed by McDevitt et al. (2002), making it the second-most common type. A defensive hate crime occurs when the offender is reacting to an intrusion upon their dominant status in society, for example, when a black family is targeted for moving into a previously all-white neighborhood, when a same-sex couple is attacked for holding hands in public, or when a woman of color is retaliated against for getting the promotion at work (Gerstenfeld, 2018).

Rowland (2019) claimed that Trump’s campaign was effective because it was affective; right-wing national populism is ruled by emotions, including rage at the establishment and the elites, fear and hatred of the other, and praise of the “savior” uniting them against a perceived common enemy. Trump’s campaign and rhetoric were exclusive and supportive of violence (Stuckey, 2020). Reports have shown that Trump directly influenced right-wing extremists who engaged in violence, planned terrorist attacks, and participated in white supremacist rallies (see e.g. Jones, 2018). This reactionary violence and terrorism were not only more likely because of Trump’s candidacy, but should have been expected and warned against. More recently, Trump’s pathologization of Asian Americans and racist anti-Asian rhetoric during the COVID-19 pandemic resulted in an increase in verbal and online abuse and violent hate crimes against them (Gover et al., 2020; Litam, 2020).

### Denigration

Compared to other forms of bias and hate violence, such as racist and anti-LGBTQ crimes, ableism and anti-disability violence has received much less attention and resources, though a renewed focus on ableist violence is fomenting in the UK in response to a lack of progress on responding to ableist violence in that country (see, for example, the British Society of Criminology’s (2022) recent conference on ableist hate crime). Trump’s ableist and denigrating speech, in most cases, appears in context as an enhancer to his more racist verbal and textual hostility. There was at least one explicitly ableist moment in his campaign—when he mocked the visible differences of reporter Serge F. Kovaleski—which ironically he defended by use of even more ableist slurs, calling Kovaleski “handicapped” and the Clinton campaign team “sick people” for even noting his ableism (Kessler, 2016). An undercurrent of ableism was thoroughly embedded in his political rhetoric up until his inauguration, when this speech act largely disappears from his lexicon until the emergence of COVID and the 2020 election campaign when he, again, reduced his antagonists and targets to disabled bodies and minds (see, for example, Cokley’s (2020) analysis of both Trump’s ableism and his opponents’ ableism used to undermine his candidacy).

In Asquith’s (2008) early taxonomy of verbal-textual hostility, denigration was largely absent from police records of what was said in reported hate crimes. This may have been an artefact of the late, explicit addition of ableist violence to the protected classes in UK hate crime legislation, or the widespread normalization by police (and victims) of ableist speech acts so that they were not perceived as important forensic evidence of ableist hate violence. Either way, despite only analyzing cases of homophobic, racist, Islamophobic and antisemitic hate crimes, Asquith noted that approximately six percent of the recorded hate speech was akin to that we have labelled as denigration in our analysis of Trump’s speeches. Serendipitously, in Trump’s speeches, this theme of verbal-textual hostility was also present in approximately six percent of the corpus. When combined with other ableist language that pathologizes, a pattern of reducing opponents to crippled bodies/minds—irrespective of their apparent lack of disability—emerges in political rhetoric. This is not unique to Trump as politicians have consistently called their opponents stupid, but as noted by Çinar et al. (2020), Trump was “abnormally extreme” in his use of ableist and denigrating language.

### Policy implications

With accumulating knowledge, there are clear policy implications. Bias incidents, including micro-aggressions and hate speech, must be recorded and responded to in order to prevent it from evolving into violence (Schweppe & Perry, 2021). Given the nature of the lethal hate crimes that have occurred in the last decade, if law enforcement is to have an impact on preventing these crimes, they must divert resources to focus on right-wing extremism and terrorism (Jones, 2018). Goodall (2009) notes that a key difference between hate speech offences and hate crimes is the audience of the crime which “does not necessarily include the victim, but is rather a separate audience who can be stirred up” (p. 213). It is this perlocutionary distinction, and the extent to which hate speech can and should be criminalized, which is perhaps one of the more contentious issues in the field of hate studies (Schweppe & Perry, 2021). Yet it seems clear that governments need to use existing regulatory frameworks to identify and decrease the amount of hostile and hateful rhetoric in the public domain, because their failure to do so normalizes and even authorizes violence (Cohen-Almagor, 2018; Waldron, 2012). The United States is an outlier among contemporary democracies for its refusal to respond to or prevent hate speech, but Trump’s campaign and presidency have clearly shown that it is time for the US to learn from their peers and develop constitutionally-sound measures for regulating hate speech. In *Brandenburg v Ohio (1969)*, the US Supreme Court laid out a narrow test for the incitement of violence that arguably can and should be applied to former President Trump and his ilk.

## Conclusion

The regulation or criminalization of hate speech has been a contentious issue for decades, yet critical criminologists, with only a few exceptions, have been painfully silent. Hate speech, like hate crime, is about social power dynamics and attempts to maintain a status quo. Given its role in the trifecta of violence and the continuum of hate, hate speech and other forms of verbal-textual hostility must be investigated and analyzed. We hope that this analysis of denigrating, deprecating, and dominating speech will inspire more research. As Cohen-Almagor and others have argued, “speech can and does inspire crime” (2018, p. 38; see also Schweppe & Perry, 2021), so it is imperative for criminologists and criminal legal scholars to contribute to the understanding of causal mechanisms and the development of prevention measures. This is particularly necessary in the context of political hate speech due the reach and impact of those in the public eye and in power.

## Data Availability

The datasets analysed during the current study are publicly available from the American Presidency Project (unaffiliated with this study), and are available from the corresponding author on reasonable request.

## Footnotes

1 Ironically, as will be demonstrated below, this reference to “small-minded people” is similar to the denigration we seek to highlight in verbal and textual hostility. While the author may have been referring to pettiness rather than intellectual impairment, their use of ableist language to denigrate people by eliding intellectual impairment with pettiness, spite, and prejudice is exactly what we highlight in this analysis of Trump’s speeches.

2 And ironically, due to the disdain of German Nazis generated by their own disdain and hatred of Jews, Bytwerk deploys deprecation to frame the outcomes of the Third Reich; perhaps one of the few instances in political discourses where deprecation was rightly applied.

3 In her more recent work on ableist violence with Thorneycroft, Asquith (2021b) has suggested that at the base of all hate speech is a niggling, not quite explicit ableism, whereby those on the margins—whether race, religious, sexuality, gender etc.—are valued on a eugenicist scale that ends with the denigration of bodies and minds that are “out of place” (Douglas, 1966).
